# Ancient Sanitary Regulations

**Published:** 1870-02

**Authors:** 


### *Ancient Sanitary Regulations.*

As early in the history of England as A. D. 1500, the scavengers, constables and wards of the city of London were ordered, "on pain of death," to see all streets and yards kept clear of dung and rubbish, and all other filthy and corrupt things. Carts went round every Monday, Wednesday and Saturday, to carry off the litter from the houses, and on each of these days twelve buckets of water were drawn for "every person," and used in cleaning their rooms and passages. — *Guildhall MSS., quoted by Froude.*

